# Ginsenoside Rg1: A Neuroprotective Natural Dammarane‐Type Triterpenoid Saponin With Anti‐Depressive Properties

**DOI:** 10.1111/cns.70150

**Published:** 2024-12-06

**Authors:** Dong Han, Zheng Zhao, Tinghui Mao, Man Gao, Xue Yang, Yan Gao

**Affiliations:** ^1^ Department of Neurology Shengjing Hospital of China Medical University Shenyang Liaoning China; ^2^ Department of Emergency Medicine Shengjing Hospital of China Medical University Shenyang Liaoning China; ^3^ Department of Organ Transplantation and Hepatobiliary Surgery The First Affiliated Hospital of China Medical University Shenyang Liaoning China; ^4^ Department of Obstetrics and Gynecology Shengjing Hospital of China Medical University Shenyang Liaoning China

**Keywords:** depression, ginsenoside Rg1, molecular docking, pharmacological effects, pharmacological network

## Abstract

**Background:**

Depression, a widespread mental disorder, presents significant risks to both physical and mental health due to its high rates of recurrence and suicide. Currently, single‐target antidepressants typically alleviate depressive symptoms or delay the progression of depression rather than cure it. Ginsenoside Rg1 is one of the main ginsenosides found in Panax ginseng roots. It improves depressive symptoms through various mechanisms, suggesting its potential as a treatment for depression.

**Materials and Methods:**

We evaluated preclinical studies to comprehensively discuss the antidepressant mechanism of ginsenoside Rg1 and review its toxicity and medicinal value. Additionally, pharmacological network and molecular docking analyses were performed to further validate the antidepressant effects of ginsenoside Rg1.

**Results:**

The antidepressant mechanism of ginsenoside Rg1 may involve various pharmacological mechanisms and pathways, such as inhibiting neuroinflammation and over‐activation of microglia, preserving nerve synapse structure, promoting neurogenesis, regulating monoamine neurotransmitter levels, inhibiting hyperfunction of the hypothalamic‐pituitary‐adrenal axis, and combatting antioxidative stress. Moreover, ginsenoside Rg1 preserves astrocyte gap junction function by regulating connexin43 protein biosynthesis and degradation, contributing to its antidepressant effect. Pharmacological network and molecular docking studies identified five targets (AKT1, STAT3, EGFR, PPARG, and HSP90AA1) as potential molecular regulatory sites of ginsenoside Rg1.

**Conclusions:**

Ginsenoside Rg1 may exert its antidepressant effects via various pharmacological mechanisms. In addition, multicenter clinical case‐control and molecular targeted studies are required to confirm both the clinical efficacy of ginsenoside Rg1 and its potential direct targets.

## Graphical abstract

Rg1 exhibits antidepressant effects through diverse mechanisms, such as anti‐inflammation, synaptic protection, neurogenesis promotion, brain‐derived neurotrophic factor release, enhancement of astrocyte gap junction function, and regulation of hypothalamic–pituitary–adrenal axis and antioxidative stress. Experimental studies validate Rg1's efficacy in depression treatment, with molecular docking analysis supporting its promising application prospects.

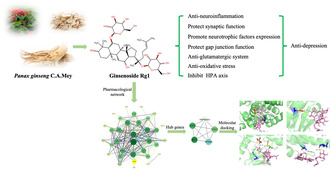

## 1 Introduction

Depression is a mental disorder that can be both spontaneous and persistent. Primary symptoms include diminished interest or pleasure, fatigue, changes in appetite and sleep patterns, difficulty concentrating, cognitive decline, and heightened suicidal tendencies, leading to elevated disability, mortality, and recurrence rates [1]. The global incidence of depression has been increasing annually. The lifetime prevalence of depression in Chinese women and men is 8.0% and 5.7%, respectively, posing a serious burden on both patients and society [2]. However, the pathophysiological mechanism of depression remains elusive. Several hypotheses exist, including the monoamine neurotransmitter, hypothalamic–pituitary–adrenal (HPA) axis, neuroplasticity, immunoinflammatory, and neurogenetic hypotheses [3, 4, 5]. Patients with depression exhibit reduced synthesis of neurotransmitters, including 5‐hydroxytryptamine (5‐HT) and dopamine, alongside excessive activation of the HPA axis, resulting in a considerable increase in glucocorticoid levels; this leads to hippocampal neuron atrophy, reduced neurogenesis in newborns and neural precursor cells, and decreased neuroplasticity [6, 7]. Various antidepressant drugs are available, including tricyclic and tetracyclic antidepressants, monoamine oxidase inhibitors, and selective 5‐HT reuptake inhibitors. However, first‐line clinical treatment yields considerable improvement in only one‐third of patients, with a latency period of several weeks to months [8]. Therefore, an urgent need for safe and rapid antidepressant therapies exists. With a long history of use, natural compounds from ethnic medicines can be used to treat depression [9].

Traditional Chinese medicine lacks the term “depression” in the ancient books; however, based on clinical manifestations, it categorizes similar conditions as “depression syndrome,” “manic,” and “Meihe Qi.” [10, 11]. *Panax ginseng* C. A. Mey serves as a medicinal remedy for spleen tonification, blood nourishment, and mind calming. In the preparation of Materia Medica, *Panax ginseng* is noted for hosting qi in the blood. Its effects include tonifying the spleen, promoting qi flourishing in the spleen, and enhancing blood biochemical activity. These actions reflect its dual tonification of both qi and blood, highlighting a heart‐spleen co‐treatment approach [12, 13]. Ginsenosides, a class of triterpenoids, are the primary medicinal components of *Panax ginseng* . More than 150 natural ginsenosides have been isolated and identified from various parts of *Panax ginseng* . Ginsenosides can be divided into oleanane and dammarane types according to the different skeletons of the ginsenoside aglycones. Among them, dammarane ginsenosides primarily contain propanaxanediol, propanaxantriol (PPT), and pseudoginsenosides as aglycones, whereas oleanane ginsenosides contain oleanolic acid [14]. Ginsenoside Rg1, a prominent PPT‐type ginsenoside, constitutes a considerable proportion of *Panax ginseng* root and exhibits diverse biological functions, including antioxidant, immunomodulatory, anti‐inflammatory, and angiogenesis‐promoting effects [13, 15]. Recently, the pharmacological activity, pharmacokinetics, and bioavailability of ginsenoside Rg1 have been studied, and new progress has been made [16]. In various neurological disease models, ginsenoside Rg1 exerts neuroprotective effects via various targets. For example, ginsenoside Rg1 improves cognitive impairment in animal models by preventing Alzheimer's disease‐related pathology, regulating synapses, and exerting anti‐inflammatory and antioxidant effects [17]. However, the antidepressant mechanisms of ginsenoside Rg1 remain elusive.

Therefore, we reviewed depression pathogenesis, current treatments, in vivo and in vitro studies, and potential mechanisms of ginsenoside Rg1 in depression treatment. Additionally, we predicted the possible anti‐depression targets of Rg1 via pharmacological network and molecular docking analyses and examined its pharmacokinetics, bioavailability, and pharmacodynamics for depression treatment potential.

## 2 Depression Pathogenesis and Approved Drugs

The pathogenesis of depression involves a complex interplay of social, psychological, and physiological factors, often accompanied by other mental disorders and chronic diseases [18]. Several theories have been proposed to explain the pathogenesis of depression [19, 20, 21]. Drug therapy remains the primary treatment for patients with depression in clinical practice, and most antidepressants target monoamine neurotransmitters [22]. However, clinical antidepressants generally have several disadvantages, including delayed onset, serious adverse reactions, high cost, susceptibility to relapse upon drug withdrawal, and easy rejection reactions by the body [23, 24]. In contrast, traditional Chinese medicine contains various chemical components with diverse pharmacological effects, facilitating the exploration of the pathogenesis of depression, developing antidepressants, and improving treatment efficacy, including synergistic management of its complications [25, 26].

### 2.1 Pathogenesis of Depression

**FIGURE 1:**
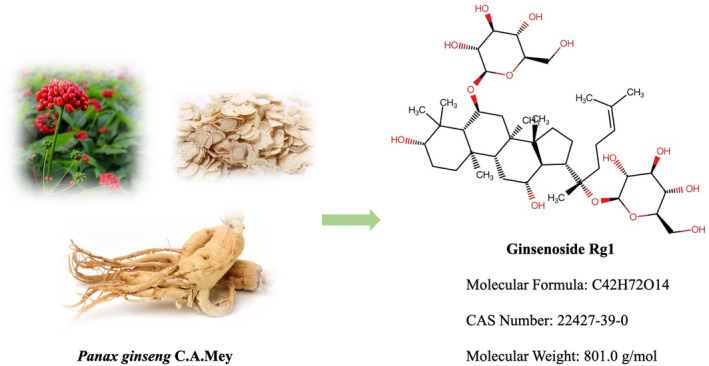
Common source, chemical structure, and molecular characteristics of ginsenoside Rg1.

Multiple mechanisms are involved in the pathogenesis of depression and primarily include abnormal expression of neurotransmitters and their receptors, dysfunction of the HPA axis, oxidative stress, mitochondrial dysfunction, neuroinflammation, decreased levels of neurotrophic factors, and imbalances in intestinal flora (Figure 1) [27, 28].

#### 2.1.1 Depression and Monoamine Neurotransmitters and Their Receptors

Increased expression of type A monoamine oxidase (MAO‐A) and decreased levels of serotonin (5‐HT) and norepinephrine (NE) in the brain are considered major factors in depression pathogenesis [29]. MAO‐A can decompose the monoamine neurotransmitters 5‐HT, NE, and dopamine (DA) and plays an important role in the onset, progression, and treatment of depressive disorders [30]. DA, 5‐HT, and other neurotransmitters play a key role in maintaining balance in brain chemistry; however, imbalances in neurotransmitter levels and abnormal signal transduction contribute to the onset and progression of depression [31].

5‐HT accumulates in nerve terminals via the serotonin transporter, which transports 5‐HT into the cytoplasm to repopulate synaptic vesicles and terminate its extracellular action [32]. 5‐HT regulates human behavior, mood, memory, and other activities, playing a key role in both the pathogenesis and treatment of depression [33]. Fuxe et al. first obtained 5‐HT neuronal cell bodies and dendrites in 1967 and found a 5‐HT reuptake mechanism in axons and nerve terminals, opening the possibility that antidepressants could act by blocking this mechanism [34]. Most clinical trials consistently indicate that decreased levels of monoamine neurotransmitters, including 5‐HT and DA, are among the primary contributors to depression [35, 36, 37]. Further elucidation of the mechanism of monoamine neurotransmitter decline revealed that the activity of indoleamine 2, 3‐dioxygenase (IDO) in the serum of patients with depression significantly increased, and the breakdown rate of tryptophan accelerated; this inhibited the metabolism of tryptophan in the 5‐HT pathway and reduced the 5‐HT concentration in the synaptic cleft, accelerating the occurrence of depression [38]. In depression, the concentration of dopamine transporter (DAT) is significantly higher than that in the general population. High DAT concentration increases DA reuptake in synaptic terminals, leading to decreased DA levels in the synaptic cleft and ultimately inducing depression in vivo [39].

Through extensive research into the pathological mechanisms of depression, it has been discovered that antidepressant drugs can rapidly increase the concentration of neurotransmitters in the synaptic cleft, although their therapeutic effects manifest relatively slowly. Therefore, the receptor hypothesis has been proposed [40]. Research has demonstrated that in the brain of patients with depression, considerable reduction in 5‐HT receptor sensitivity and postsynaptic membrane quantity exists [41, 42]. These findings suggest that a small number of antidepressants can be clinically administered by changing the adrenaline and 5‐HT receptor sensitivity, thereby increasing 5‐HT or NE levels and improving depressive symptoms [43]. Recent research indicates that blocking the β2 receptor specificity on presynaptic membranes reduces the body's ability to adjust feedback of NE and 5‐HT, resulting in increased levels of NE and 5‐HT in the synaptic cleft, thereby enhancing the antidepressant effect [44, 45]. Therefore, neurotransmitter or receptor disorders may lead to depression.

#### 2.1.2 Depression and Hypothalamic–Pituitary–Adrenal Axis Disorder

The HPA axis comprises the hypothalamus, pituitary glands, and adrenal glands, which play important regulatory roles in the body [46]. From primitive organisms to humans, the HPA axis plays a crucial role in various biological processes [47].

Activation of the HPA axis and its cascade effect can occur following physical or psychological stress. It begins with the hypothalamus secreting corticotropin‐releasing hormone (CRH), which stimulates the pituitary gland to release adrenocorticotrophic hormone (ACTH). ACTH subsequently stimulates the adrenal cortex to release cortisol (COR), leading to various physiological reactions in the sympathetic nervous system [48, 49]. This response is terminated by a negative feedback loop after the stressor disappears. In this cycle, decreased COR levels and inhibition of the further release of ACTH and CRH play key roles in regulating mood in the hippocampus [50]. Downregulation of glucocorticoid receptor (GR) protein expression in the hippocampus leads to reduced functionality, impairing its negative feedback regulation on the HPA axis. Consequently, the failure of negative feedback allows the HPA axis to remain hyperactive. Subsequently, the function of the hippocampal 5‐HT system decreases owing to excessive COR levels, and the decreased expression of various neurotrophins leads to the degeneration of hippocampal neurons, ultimately leading to depression [51].

Different types of antidepressants, including monoamine oxidase inhibitors (MAOIs), tricyclic drugs, and selective serotonin reuptake inhibitors (SSRIs), can regulate HPA axis activity in humans and animals via different mechanisms [52]. Regulation of the HPA axis is primarily focused on the following aspects [53, 54, 55]: (i) reduction of HPA axis activity, (ii) improvement of glucocorticoid resistance by enhancing GR function, and (iii) enhancing the regulatory function of the HPA axis. In recent years, other types of drugs have been found to exert antidepressant effects.

#### 2.1.3 Depression and Inflammatory Response

Over the past 20 years, an increasing number of studies have confirmed that inflammatory responses are involved in the pathogenesis of depression [56]. Maes et al. [57] first found that the plasma level of interleukin‐1 (IL‐1) increases in patients with severe depression, and the abnormal expression of this protein is often regarded as an important sign of the body's inflammatory state. Therefore, the occurrence of depression may be closely related to the body's inflammatory response. Subsequent studies reveal that individuals with depression often exhibit elevated levels of tumor necrosis factor‐alpha (TNF‐α) and other inflammatory factors. Following antidepressant treatment, there is a reduction in anti‐inflammatory factor levels in the body [8, 58]. Inflammation affects the synthesis of central monoamine transmitters. Chronic stress and other factors can cause the activation of microglia and subsequently produce various pro‐inflammatory factors, such as IL‐1, IL‐6, interferon‐γ (IFN‐γ), and TNF‐α. These cytokines activate IDO and GTP‐cyclohydrolase 1 (GTP‐CH1) via signal transducer and activator of transcription 1a (STAT1a), interferon regulatory factor 1 (IRF‐1), nuclear factor kappa B (NF‐κB), and p38 mitogen‐activated protein kinase (MAPK) pathways [59]. Sustained activation of IDO in the brain leads to the breakdown of tryptophan into kynurenine, which is further metabolized to 3‐hydroxykynurenine (3‐HK) and quinolinic acid. Both of these are neuroactive glutamate compounds that activate the glutamate N‐methyl‐D‐aspartate receptor (NMDAR), leading to oxidative stress [60]. Abnormal activation of NMDAR can inhibit the production of brain‐derived neurotrophic factor (BDNF) and neurogenesis, resulting in brain volume changes, dendritic atrophy, and synaptic loss. In addition, GTP‐CH1 activation leads to a decrease in the synthesis of tetrahydrobiopterin (BH4), an essential cofactor for DA and 5‐HT biosynthesis. Inflammatory cytokines activate GTP‐CH1 and promote the oxidation of BH4, affecting the synthesis of DA and 5‐HT [61]. Moreover, inflammatory factors can lead to HPA axis activation, which increases tryptophan 2, 3‐dioxygenase (TDO) activity, degrades tryptophan, and decreases 5‐HT production [62]. Thus, the inflammatory response may be an important mechanism in the onset of depression.

#### 2.1.4 Depression and Oxidative Stress

Oxidative stress occurs when the body produces an excess of highly reactive free radicals, including reactive oxygen species (ROS) and reactive nitrogen species, when the body is subjected to various harmful stimuli, surpassing the body's antioxidant capacity. This imbalance leads to oxidative damage to tissues [63]. ROS, primarily produced in the mitochondria, include superoxide anions, hydroxyl radicals, and hydrogen peroxide. These ROS can damage multiple components of brain tissue cell membranes, ultimately leading to neuronal damage [64]. The hypothalamus, hippocampus, and frontal cortex are sensitive to various stress stimuli and are closely associated with affective disorders [65]. Oxidative stress plays a key role in the pathophysiology of depression, including a decrease in the defense capacity of the antioxidant system, generation of peroxidative damage, and secondary autoimmune responses [66]. Lipid peroxidation of the cell membrane primarily occurs in the phospholipid bilayer [67]. The role of the endogenous antioxidant system in chronic stress‐induced neuronal injury has also been investigated. The levels of lipid peroxidation, total antioxidant capacity, catalase, and glutathione oxidase in the frontal cortex, hippocampus, and striatum were analyzed in rats exposed to chronic unpredictable mild stress (CUMS). The level of the lipid peroxidation product malondialdehyde (MDA) increased. Decreased total antioxidant capacity, glutathione oxidase, and catalase activities suggest reduced endogenous antioxidant system function. This decline can lead to lipid peroxidation, potentially leading to depression [68].

Apoptosis is an autonomous programmed cell death process, whereas cell necrosis is a type of cell death that can induce inflammation and may be accompanied by cell damage [69]. ROS can mediate cell necrosis via endogenous and exogenous pathways, which are associated with mitochondrial dysfunction and cell death receptor pathways, respectively [70]. The rat depression model is accompanied by increased TNF expression in the brain tissue, leading to nerve cell necrosis. Therefore, regulating the expression level of TNFs in brain tissue may contribute to the treatment of depression [71, 72]. ROS, acting as a key secondary messenger, activates NF‐κB and MAPK pathways to regulate cell apoptosis and generate proinflammatory molecules, ultimately leading to cell death and immune system activation. This intricate cascade involves transcription factors, such as Nrf2 and NF‐κB, influenced by environmental factors, contributing to the complexity of depression pathogenesis [73]. Caspases are a group of aspartic proteolytic enzymes with similar structures that exist in the cytoplasm and can cleave Asp‐X peptide bonds in proteins [74]. This cascade reaction involves the intersection of multiple apoptotic pathways, ultimately leading to apoptosis [75]. Consequently, caspases promote an increase in ROS levels [76]. Although caspase‐3 does not induce cell death, it can inhibit the function of apoptotic proteins and proteasomes, indicating that caspase activation is limited by these molecular mechanisms [77]. Hüttemann et al. [78] demonstrated high cytochrome C (Cyt‐C) expression in the hippocampus of rats in the depression model group. This increase may be due to Cyt‐C release into the cytoplasm after cell stimulation, activating the caspase pathway and resulting in cell apoptosis or necrosis, thereby leading to depression‐like behavior [78]. In summary, numerous studies have shown that oxidative stress is closely related to depression and is involved in its pathogenesis via mitochondrial damage, upregulation of oxygen‐free radical levels, and reduction of antioxidant levels.

#### 2.1.5 Depression and Mitochondrial Dysfunction

Mitochondria are the “power factories” of cells, participating in complex physiological activities, such as energy metabolism, cell survival, and nervous system development [79]. Patients with major depression commonly exhibit hyperactivity of the glucose anaerobic oxidation pathway and lactic acid accumulation in the central nervous system. This shift from aerobic to anaerobic oxidation pathways in glucose metabolism is linked to mitochondrial dysfunction [80, 81]. Chu et al. [82] observed significant increases in lactate/N‐acetyl‐aspartate and lactate/total creatine ratios in the caudate nucleus and anterior cingulate cortex of patients with bipolar depression. Additionally, offspring exposed to maternal stress during pregnancy and subsequent depression exhibited elevated lactic acid concentrations in the prefrontal cortex (PFC) of rats [83]. Therefore, changes in glucose metabolism in the central nervous system from aerobic to anaerobic oxidation pathways, along with the hyperactivity of the glucose anaerobic oxidation pathway, contribute to mitochondrial energy metabolism disorders, leading to depression.

Mitochondrial biogenesis is a physiological response of cells to external stress, which increases energy demand. Peroxisome proliferator‐activated receptor‐γ coactivator (*PGC‐1α*) is a key gene that induces mitochondrial biogenesis [84]. Ryan, Patterson, McLoughlin [85] observed reduced expression of *PGC‐1α* in whole blood samples from patients with major depression. Alcocer‐Gómez et al. [86] also found that the expression of *PGC‐1α* and its downstream target genes, such as nuclear respiratory factor 1 (*NRF1*) and mitochondrial transcription factor A (*TFAM*), is downregulated in blood monocytes of patients with depression, indicating that mitochondrial biogenesis disorder caused by low PGC‐1α expression may be a pathogenesis of depression.

Mitophagy includes the PTEN‐induced putative kinase 1 (PINK1)/Parkin and mitophagy receptor‐mediated pathways. The PINK1/Parkin pathway is a ubiquitin (Ub)‐dependent autophagy pathway in damaged mitochondria. PINK1 promotes mitophagy by recruiting and phosphorylating Parkin and Ub to the depolarized mitochondrial outer membrane [87]. Liu et al. [88] suggested that maternal stress during pregnancy reduces PINK1 expression in the hippocampus of offspring rats, leading to abnormal mitophagy. This mechanism may play a crucial role in offspring depression. Agnihotri et al. [89] also indicated that mitochondrial clearance disorders caused by PINK1 deficiency reduced the threshold of depression‐like behaviors induced by chronic restraint stress in mice, indicating that the inhibition of mitophagy may be involved in the pathogenesis of depression.

Mitofusin (Mfn) is a transmembrane protein with guanosine triphosphatase activity on the outer mitochondrial membrane. It serves as a crucial indicator of mitochondrial fusion [90]. Goetzl et al. [91] observed a decrease in Mfn‐2 expression in neuron‐derived extracellular vesicles in patients with major depression, which normalized following 8 weeks of treatment with a selective 5‐HT reuptake inhibitor. Liu and Zhou [92] also found that Mfn‐1 and Mfn‐2 expression decreased in the brains of rats with chronic mild unpredictable stress and glucocorticoid‐induced depression. These studies suggest that abnormalities in mitochondrial fusion may be involved in depression.

Mitochondrial dysfunction is closely associated with depression development. Disorders in mitochondrial energy metabolism and mitochondrial quality control can be observed in patients and animal models of depression. Antidepressant treatments targeting the mitochondria have certain drug development prospects.

#### 2.1.6 Depression and Neurotrophic Factors

BDNF is an important neurotrophic factor found in the human brain that is primarily secreted by neurons and glial cells [93]. It plays an important role in the survival and differentiation of neurons, axon growth, synaptic plasticity, angiogenesis, learning, emotional regulation, and memory [94]. Decreased BDNF levels in the hippocampus and PFC of patients with depression are associated with reduced hippocampal volume, decreased neuronal proliferation in the CA3 region, and increased neuronal apoptosis in the dentate gyrus region [95]. Na et al. [96] compared BDNF promoter methylation and cortical thickness in patients with recurrent depression and healthy individuals and found that BDNF promoter methylation increased in the PFC, left occipital cortex, and precuneus of patients with major depression, along with thinner cortices in these regions. These studies suggest that BDNF is closely related to depression. Decreased BDNF levels may reduce the synaptic plasticity of neurons in relevant brain regions, resulting in a decline in cognitive function, weakening of emotional regulation ability, and continuous accumulation of negative emotions, thereby influencing the onset and progression of depression.

Numerous BDNF‐related studies have focused extensively on the relationship between the precursor of BDNF (pro‐BDNF) and mature BDNF (mBDNF) and depression [97]. The amount of pro‐BDNF cleavage is related to the occurrence of depression. Following ketamine treatment, the cleavage of pro‐BDNF in the hippocampus significantly increased, suggesting that pro‐BDNF is related to the occurrence of depression and may be an indicator of antidepressant efficacy [98]. mBDNF is derived from proBDNF. Zhang et al. suggested that the mBDNF expression level in the hippocampal CA1 region is reduced in patients with depression. Following antidepressant treatment, mBDNF levels in the prefrontal lobe of patients increase [99, 100]. Bus et al. [101] further indicated that mBDNF may play a role in the development of depression by modulating the plasticity of neurons in the dentate gyrus of the hippocampus.

Studies on the neurotrophic factor hypothesis of depression indicate that the tissue‐type plasminogen activator (tPA) plasminogen system plays an important role in the pathophysiological process of depression by regulating the transformation of pro‐BDNF to mBDNF [102]. Madani, Nef, Vassalli [103] have shown that the tPA pathway plays a key role in the pathophysiology of mood disorders. The animal experiments of Pawlak et al. [104] showed a rapid increase in tPA levels in the amygdala of mice subjected to unpredictable stress. When mice are repeatedly subjected to stress, the molecular structure of tPA in the brain tissue is destroyed, and mice exhibit anxiety and depression [105]. The above studies suggest that tPA is an important factor affecting mood, and a decrease in its expression level may lead to adverse mood states, potentially culminating in depression.

The neurotrophic factor theory largely explains the mechanism of the occurrence and development of depression. BDNF, a major member of this family, has garnered the attention of clinicians in depression, making its signaling pathway a focal point in antidepressant research.

#### 2.1.7 Gap Junction Function of Hippocampal Astrocytes

The hippocampus is an important part of the limbic system involved in the regulation of learning, memory, and emotional activity and plays a crucial role in the evolution of depressive mood disorders. The hippocampus is rich in astrocytes. Depression can lead to changes in hippocampal volume and astrocytes [106, 107]. Cell counting studies on postmortem brain tissue from patients with depression demonstrated that the number of astrocytes in the dorsolateral PFC, anterior cingulate cortex, subgenual PFC, and other brain regions is reduced [108, 109]. In addition, experimental animal studies have shown that chronic stress leads to a decrease in astrocyte density in the hippocampus and PFC [110]. Astrocytes are widely connected via gap junctions and form functional syncytia [111]. Gap junctions enable astrocytes to form a three‐dimensional network structure, also known as an astroglial network. Gap junction channels are formed by docking a hemichannel on the cell membrane of two adjacent cells. Each hemichannel consists of six connexin subunits that form a hexamer structure. It is a channel facilitating the direct exchange of materials and information between cells. Therefore, the astrocyte network plays an important role in processing and integrating information from numerous neurons, regulating neural transmission, supplying energy to neurons over long distances, and buffering ion and neurotransmitter concentrations to protect neurons [112]. Gap junction dysfunction of astrocytes in the hippocampus leads to functional changes in neurons in this area, consequently resulting in the dysfunction of other emotion‐related brain areas, ultimately contributing to the onset of depression [113, 114].

Recent studies have confirmed that the dysfunction of connexin43 protein and its component channels in astrocytes may be closely related to depression [115]. Miguel‐Hidalgo et al. [116] studied the prefrontal lobe of brain samples derived from autopsy and proposed that prefrontal lobe dysfunction in patients with depression is accompanied by a decrease in connexin43 level. Thus, the pathological changes of mental and behavioral abnormalities may involve dysfunction in gap junctions or changes in cell communication based on semi‐channels. Shen et al. [117] demonstrated that genistein improved depression by targeting connexin43 to inhibit miRNA‐221/222 expression. Following genistein treatment, miRNA‐221/222 expression significantly decreased, whereas connexin43 expression was upregulated. These results suggest that genistein may improve depression by inhibiting miRNA‐221/222 or increasing connexin43 expression. In summary, connexin43 protein and its component channels in astrocytes of the cortex or frontal lobe play a crucial role in depression onset. In contrast to previous drug developments that primarily target neurons, hippocampal astrocytes are expected to become new therapeutic targets.

#### 2.1.8 Intestinal Flora

Behavior, emotion, and cognition in the central nervous system are linked to the function and microecology of the gastrointestinal tract through the microbiome–gut–brain axis [118], often accompanied by changes in the intestinal flora in different animal models of depression [119]. Spearman's correlation coefficient analysis reveals a connection between changes in intestinal flora and depression‐related behaviors and indicators [120]. CUMS‐induced depression in mouse models changes the composition of the intestinal flora, primarily manifesting as changes in the diversity and richness of the intestinal flora [121]. Jianguo et al. [122] investigated the potential correlation between depressive‐like symptoms and changes in fecal metabolites in rats and found that changes in the gut microbiota may affect depressive‐like symptoms in CUMS rats via intestinal metabolites. Furthermore, the transplantation of fecal microbiota from patients with depression into germ‐free rodents led to changes in depressive‐like behavior compared to specific pathogen‐free (SPF) rats [123]. The disturbance of Firmicutes in the intestinal flora of depression‐like macaques is closely related to depressive‐like behaviors [124]. These studies suggest a close relationship between intestinal flora imbalances and depression.

Dysbiosis of the gut microbiota can interfere with RNA transcription and post‐transcriptional regulation by altering gene expression [125]. The gut microbiota regulates brain gene transcription, primarily manifesting as the differential hippocampal mRNA expression in GF mice compared to abnormal expression in SPF mice. This highlights the considerable impact of gut microbiota on hippocampal mRNA expression levels in mice and subsequent hippocampal function [126, 127]. Long non‐coding RNA (lncRNA) expression in the hippocampus of GF mice is different from that in SPF mice, suggesting that the intestinal flora has a regulatory effect on mouse hippocampal lncRNAs [127]. Most differentially expressed proteins in the hippocampus of GF mice are related to the cAMP response element‐binding protein (CREB) signaling pathway [128], which serves as a nexus for several cell signaling pathways associated with depression onset and antidepressant effects. The intestinal flora may affect the normal function of CREB by regulating lncRNA expression in the hippocampus of mice, thereby regulating depression. The gut microbiota can also regulate the expression levels of circular RNA (circRNA), which are important for the onset and development of depression. The relative abundances of Firmicutes, Proteobacteria, and Bacteroides were altered in the gut of CUMS‐induced depression‐like mice, which correlated with circHIPK2 expression. Fecal microbiota transplantation in NOD‐like receptor protein 3 (NLRP3)‐knockout mice can improve the dysbiosis of CUMS mice, regulate circHIPK2 expression, reverse the dysfunction of astrocytes, and improve the depression‐like behavior of CUMS mice [129].

The gut microbiota participates in the regulation of various cellular signal transduction pathways and plays an important regulatory role in depression. For example, *Lactobacillus casei* intervention reversed CUMS‐induced changes in the gut microbiota structure in rats by activating the BDNF‐tyrosine kinase receptor B (TrkB) signaling pathway and inhibiting the phosphorylation of extracellular signal‐regulated kinase 1/2(EKR1/2) and p38 in the frontal cortex. Thus, it can effectively improve CUMS‐induced depression‐like behavior in rats [130]. *Lactobacillus reukii* NK33, *Bifidobacterium adolescentis* NK98, and *Lactobacillus gasseri* NK109 can reverse the intestinal flora imbalance induced by *Escherichia coli* K1, inhibit NF‐κB activation in the hippocampus of mice, effectively prevent the occurrence of inflammatory responses in the brain, and reduce K1‐induced cognitive impairment and depression‐like behaviors [131, 132]. *Lactobacillus delbruecii* treatment reverses lipopolysaccharide (LPS)‐induced depression in mice by reducing Toll‐like receptor 4 (TLR4) mRNA and protein expression in the mouse brain, thereby inhibiting TLR4 signaling, effectively reducing the inflammatory response in the brain, and exerting an antidepressant effect [133].

In conclusion, gut microbiota is closely related to depression onset. The gut microbiota regulates the function of the hippocampus, microglia, and astrocytes by regulating coding RNA, non‐coding RNA, and various signaling pathways, and also regulates BDNF expression and the immune inflammatory response related to depression, thus affecting the onset and development of depression.

### 2.2 Approved Anti‐Depression Drugs

Recently, rapid progress has been made in the study of depression pathogenesis and the identification of specific drug treatment targets [134]. Antidepressants can be roughly divided into the following categories: MAOIs, tricyclic antidepressants (TCAs), tetracyclic antidepressants, SSRIs, 5‐HT and NE reuptake inhibitors (SNaRIs), and antidepressant traditional Chinese medicine [25, 135, 136].

MAOIs were the earliest drugs studied to treat depressive behavior, including non‐selective MAOI and A‐type MAOI. The former primarily affects monoamine oxidase (MAO), inhibits its activity, and increases monoamine neurotransmitter content in the nervous system, achieving the effect of treating depressive behaviors. However, owing to its severe side effects, it is rarely used in clinical practice. An A‐type MAOI can selectively inhibit MAO‐A, and its inhibitory effect is reversible, resulting in fewer toxic side effects and adverse reactions [137]. It is widely used in clinical practice, and representative drugs include moclobemide and toloxazone. Among these, moclobemide has a rapid onset of action and recovery of MAO‐A activity, making it especially suitable for depression with psychomotor inhibition, cognitive impairment, and depression in older individuals [138].

TCAs, also known as first‐generation monoamine reuptake inhibitors, inhibit the reuptake of presynaptic membrane neurotransmitters and have anticholinergic effects. TCAs, such as amitriptyline, protiline, and doxepin, have notable therapeutic efficacy across various types of depression [139]. Tetracyclic antidepressants primarily affect the monoamine neurotransmitter uptake by selectively blocking the adrenal α2 receptors in the presynaptic membrane, NE concentration in the synaptic cleft, and brain 5‐HT receptor to relieve mental depression, thus achieving antidepressant effects [140].

The primary mechanism of action of SSRIs is to specifically act on the 5‐HT transporter, inhibit the reuptake of 5‐HT, increase 5‐HT content in the synaptic cleft, and subsequently improve the function of 5‐HT [141]. SSRIs exhibit a broad antidepressant spectrum, high applicability, and high bioavailability, making great progress in drug safety. The representative drugs are sertraline, citalopram, fluoxetine, paroxetine, and fluvoxamine [142].

SNaRIs are characterized by a dual‐action mechanism that specifically acts on 5‐HT and NE. Their therapeutic effect on depressive behavior is primarily achieved by changing the reuptake function of both neurotransmitters. This class of drugs has a substantial therapeutic effect in patients with all types of depression and dose adherence. SNaRIs, such as venlafaxine and duloxetine, have poor affinity for the receptor site, greatly improving the safety and tolerability of the drug [143]. Nomifensine, bupropion, and methylphenidate are NE and DA reuptake inhibitors, respectively, and are used in clinical practice. They effectively treat depression by inhibiting the reuptake of NE and improving the therapeutic effects of NE and DA [144].

Newly developed antidepressants are important supplements to traditional antidepressants. Although the mechanisms of action of these new antidepressants remain under investigation, their roles in clinical practice cannot be ignored. Esketamine, a single dextramer of ketamine, has a higher affinity for NMDA and causes fewer adverse reactions than ketamine [145]. On March 5, 2019, the Food and Drug Administration approved the use of Janssen's esketamine nasal spray for treatment‐resistant depression in adults. Vilazodone, oxypiperidone hydrochloride, tandospirone, buspirone, and gepirone are partial 5‐HT_1A_ receptor agonists and SSRIs that are characterized by strong efficacy, rapid onset, and low incidence of adverse reactions [146].

Melatonin is an amine hormone produced by the pineal glands of mammals and humans and is derived from 5‐HT. It has several physiological functions, including sleep promotion, circadian rhythm regulation, anti‐aging, immunity regulation, and anti‐tumor effects [147]. Agomelatine, a representative drug of this class, has a positive phase adjustment effect on sleep, induces sleep‐phase advancement, and causes melatonin‐like effects [148]. Agomelatine was approved for marketing in 2009 and is currently recommended as a first‐line treatment for patients with depression and sleep disorders.

Vortioxetine, an antidepressant drug with multiple mechanisms of action, was jointly developed by Lundbeck and Takeda [149]. In addition to selectively inhibiting 5‐HT reuptake, it activates 5‐HT1A receptors, partially activates 5‐HT1B receptors, antagonizes 5‐HT3 receptors, and inhibits 5‐HT7 and 5‐HT1D receptors. It is safe, well‐tolerated, has a low incidence of adverse reactions, and significantly affects recurrent depression [150].

Brexanolone is a novel allosteric modulator of gamma‐aminobutyric acid type A (GABAA) receptors, which modulates their function both within and outside synapses, thereby restoring the balance between inhibitory and excitatory receptors in the brain [151]. In phase III clinical trials, brexanolone significantly improved depressive symptoms in patients [152]. Currently, this commercially available drug is primarily administered via intravenous infusion, whereas the oral formulation, SAGE‐217, is in early clinical trials [153].

In conclusion, synthetic second‐generation antidepressants are the first choice for the clinical treatment of depression; however, they have some limitations. Antidepressants have a long onset time, single target, and are prone to toxic side effects and drug resistance, which affect the treatment effect of depression. However, the newly developed antidepressant drugs are expensive and cannot meet the daily needs of the depressed population. Most natural drugs have multiple components, targets, mild effects, and relatively few adverse reactions. Recently, natural drugs have become a new research and development hotspot and may provide new directions for the treatment of depression in the near future.

## 3 Ginsenoside RG1

The main active component of the *Panax* genus is triterpene saponins, also known as ginsenosides, widely found in the roots, leaves, stems, flower buds, and berries. Ginsenoside content is listed as a marker for quality evaluation of ginseng in Chinese Pharmacopeia. To date, more than 150 ginsenosides have been isolated and identified [154]. They are roughly classified into three groups based on structure: (i) protopanaxadiol type (Rb1, Rb2, Rb3, Rc, Rd., Rg3, CK, Rk1, Rh3, and Rh2); (ii) protopanaxatriol type (Re, Rf, Rg1, Rg2, and Rh1); and (iii) oleanolic acid type (Ro) [14, 154]. Recent studies have confirmed the neuroprotective activity of ginsenosides. For example, ginsenosides Rh1 and Rh3 improve scopolamine‐induced memory impairment [155]. Ginsenoside Rh1 increases the hippocampal dentate gyrus excitability in mice, improves the survival rate of dentate gyrus cells, upregulates BDNF expression, and reduces oxidative stress in the cerebral cortex and hippocampus [156]. Wang et al. [157] reported that ginsenosides Rb1, Rb2, and Rb3 prevent memory impairment by protecting pyramidal and gamma‐aminobutyric acid (GABA) neurons, reducing acute excitotoxicity, and delaying the damage caused by kainic acid.

Among the various ginsenosides, Rg1 is an active ingredient with neuroprotective effects against neurodegeneration. Ginsenoside Rg1 improves Alzheimer's disease caused by neurofibrillary tangles. Rg1 also enhances working and spatial memory in rats and improves cognitive dysfunction induced by repeated alcohol consumption in mice [158]. Zhang et al. [159] found that APP/PS1 mice treated with ginsenoside Rg1 showed significantly improved cognitive dysfunction and neuronal damage. In addition, ginsenoside Rg1 alleviates high mortality and behavioral disorders in 1‐methyl‐4‐phenyl‐1,2,3,6‐tetrahydropyridine‐induced Parkinson's disease (PD) mice [160]. However, the therapeutic effects of ginsenoside Rg1 in depression have not yet been elucidated.

### 3.1 Physicochemical Properties and Distribution of Ginsenoside Rg1

**FIGURE 2:**
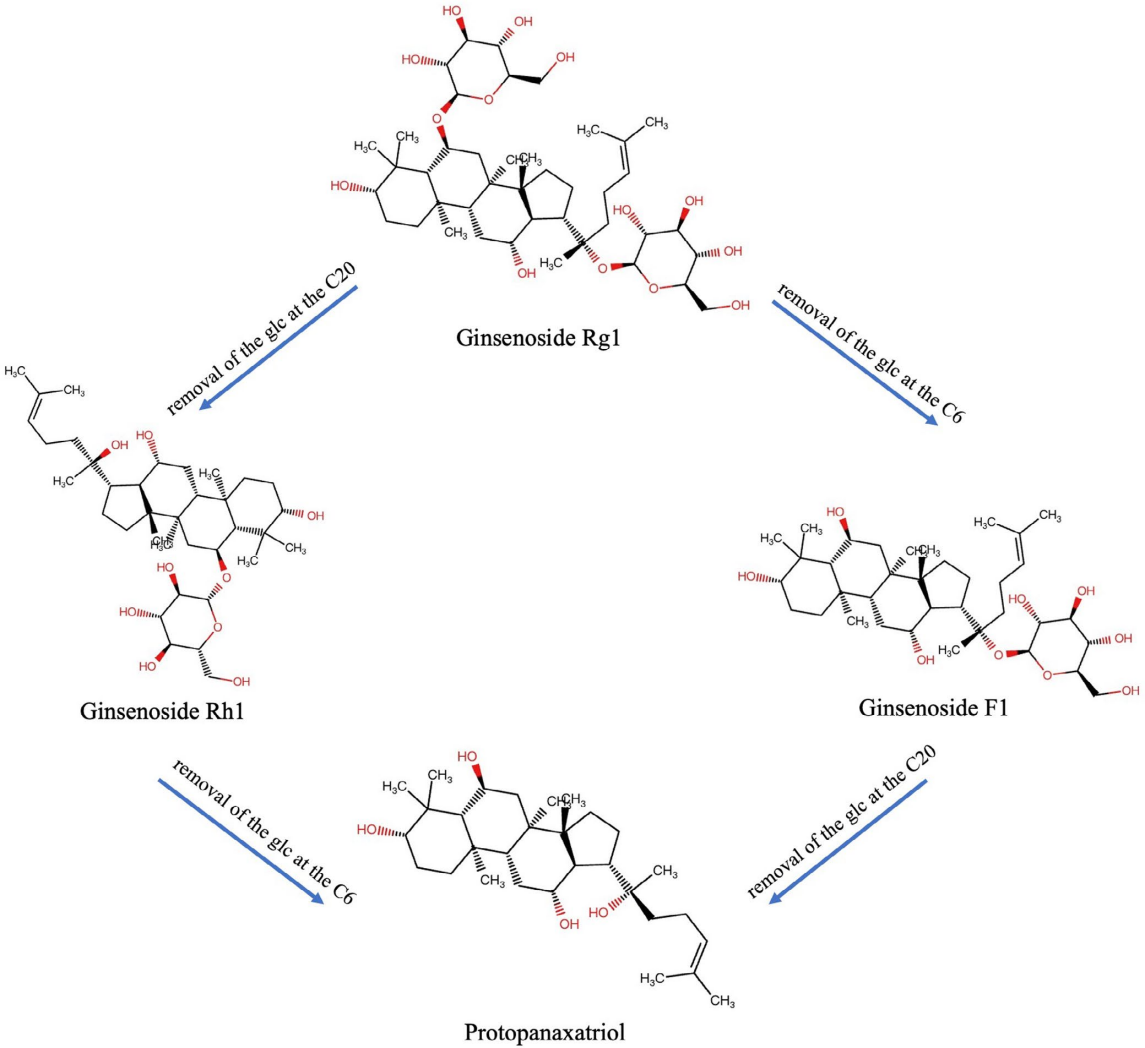
Deglycosylation of ginsenoside Rg1.

**FIGURE 3:**
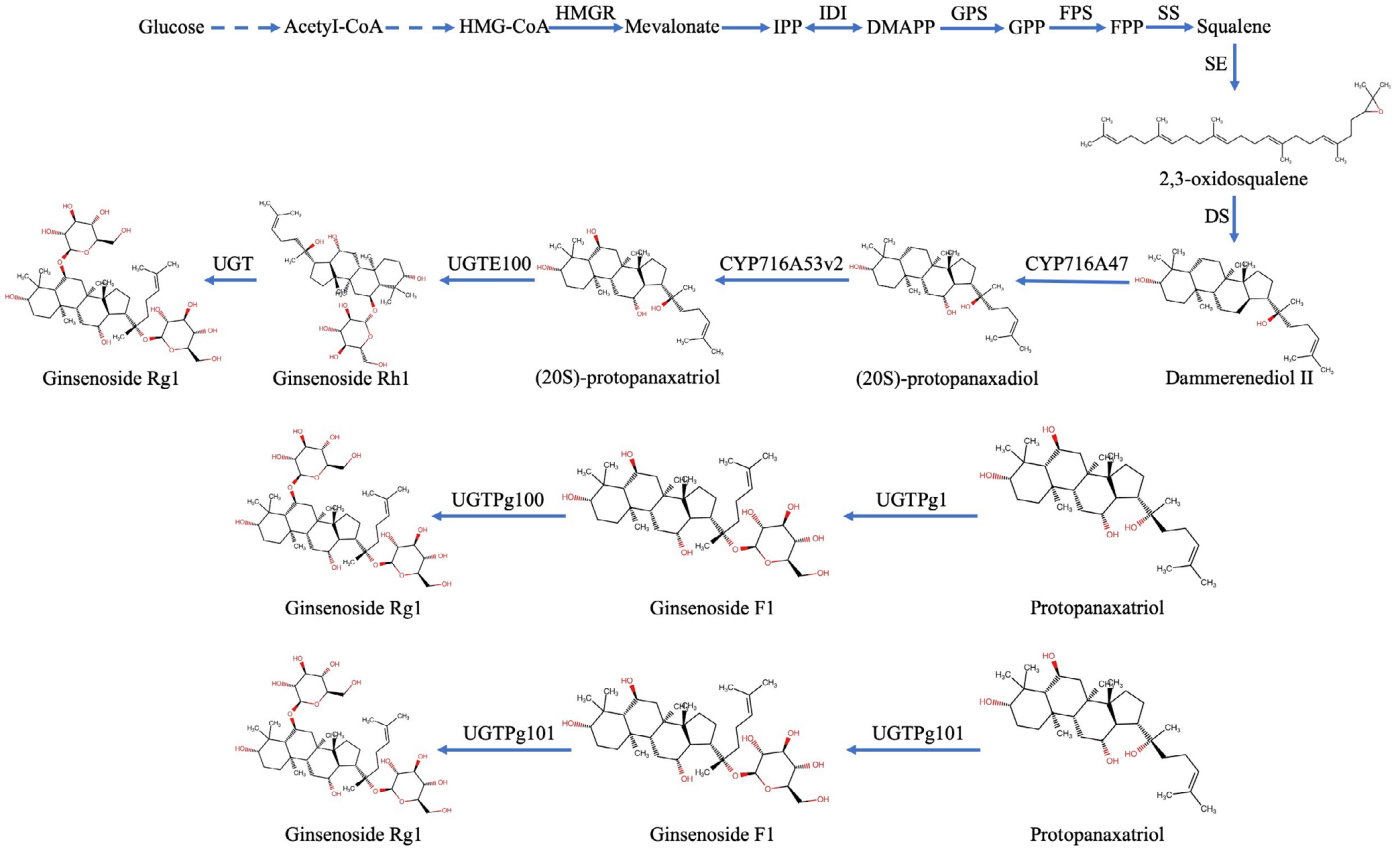
Biosynthesis pathway of ginsenoside Rg1. (A) Biosynthetic pathway of ginsenoside Rg1 in the Panax plant with key catalytic enzymes. (B) Proposed pathway for Rg1 biosynthesis based on the functions of the three UGTs, UGTPg1, UGTPg100, and UGTPg101. DMAPP, dimethylallyl diphosphate; DS, dammarenediol synthase; FPP, farnesyl diphosphate; FPS, farnesyl diphosphate synthase; GPP, geranyl diphosphate; GPS, geranyl diphosphate synthase; HMGR, 3‐hydroxy‐3‐methylglutaryl‐CoA reductase;IPP, isopentenyl diphosphate; SE, squalene epoxidase; SS, squalene synthase; UGT, UDP‐glycosyltransferase; β‐AS, β‐amyrin synthase.

The chemical structure of ginsenoside Rg1 (C_42_H_72_O_14_) is shown in Figure 2. It is a white powder with a molecular weight of 801.0. It is easily soluble in water, methanol, and ethanol and remains insoluble in ether and benzene [161]. Ginsenoside Rg1 is present in the roots, stems, leaves, flowers, and fruits of ginseng. The monomeric ginsenoside content in the extracts of different parts of ginseng is shown in Table 1 [162, 163]. The metabolites produced by the hydrolysis of ginsenoside Rg1 are ginsenosides F1 and Rh1 [164, 165]. Lee et al. [166] confirmed that intestinal flora in rats with colitis decompose ginsenoside Rg1 into intermediate ginsenosides Rh1 and Fl, ultimately metabolizing it into 20(S)‐protopanaxriol (Figure 3).

**TABLE 1:** Monomeric saponins content in extracts from different parts of *Panax ginseng* (%).

Structures	Rg1	Rg3	Rb1	Rb2	Rb3	Rc	Rd	Re	F1
Root extract	3.24	0.71	16.95	8.40	1.48	9.75	6.77	7.54	—
Stem and leaf extract	5.92	0.70	1.47	3.49	3.73	2.72	6.84	14.05	2.16
Flower extract	2.72	0.48	1.79	1.52	1.94	1.48	4.80	7.48	1.09
Fruit extract	1.10	1/34	0.33	3.13	0.62	2.75	7.44	15.22	0.44

### 3.2 Availability and Extraction of Ginsenoside Rg1

Ginsenoside Rg1, a marker of ginsenoside quality, is a challenging compound to isolate owing to its small molecule size. Various extraction methods, including enzymatic extraction, microwave‐assisted extraction (MAE), and pulse electrical extraction, can be employed [167, 168, 169]. Its high hydrophobicity complicates isolation. Although the enzymatic extraction method offers a short duration and high efficiency, it necessitates strict conditions. MAE, though yielding high extraction rates with low energy consumption, poses challenges in operational process control. The pulsed electrical extraction method consumes low energy; however, it remains in the development stage. High‐performance liquid chromatography (HPLC) has been widely used for the separation and analysis of ginsenosides owing to its good stability and high extraction rates. Most HPLC methods employ a C18 stationary phase with gradient solutions. Owing to the lack of pigments in ginsenosides, the detection wavelength typically falls within the ultraviolet (UV) range, approximately 196–205 nm. In addition, HPLC can be used to quantitatively determine neutral, acidic, and other non‐hydrophilic components [170, 171]. The content of ginsenoside Rg1 in dried ginseng root was 0.22% ± 0.02%, as determined by HPLC [172]. Zhang et al. [173] used the HPLC isospecific gradient method to separate the ginsenosides (Re, Rh1, Rg1, and Rg2) quickly and simply. In particular, ginsenosides Re and Rg1, which are difficult to separate, also exhibited a considerable separation effect. Simultaneously, the analysis was shortened to one‐fourth of the original duration. Optimizing gradient elution conditions ensures complete separation of ginsenosides Rg1 and Rf. Compared with the existing ginsenoside analysis methods, this approach eliminates the need for complex solid‐phase extraction processes. Wu et al. [174] used HPLC‐tandem mass spectrometry to separate ginsenoside Rg1, and the recovery rate reached 91.5%–92.4%. Lin et al. [175] developed an ionic liquid‐based ultrasound‐assisted extraction (ILUAE) method to extract ginsenoside Rg1 from ginseng roots. Compared to conventional ultrasound‐assisted extraction, the efficiency of this method increased by 3.16 times, and the extraction time was shortened by 33%. These results suggest that the ILUAE can significantly improve the extraction efficiency of ginsenoside Rg1. Furthermore, the Sepbox automated multi‐step preparation separation system and multi‐zone multi‐column dynamic series technology have been used for the isolation and extraction of various ginsenosides, including ginsenoside Rg1, from *Panax ginseng* and *Panax notoginseng* [176, 177].

The ginsenoside Rg1 content was significantly different in ginseng roots collected during different seasons and obtained using different processing methods. The level of ginsenoside Rg1 in the ginsenoside B‐panaxatriol group was the highest at the end of March and gradually decreased in April; however, the content of ginsenosides Rg1 and Re remained between 80% and 84% year‐round [178]. Rg1 content decreased during steaming. Its concentration in fresh and steamed ginseng was 3.272 ± 1.823 and 2.919 ± 1.726 mg/g, respectively [179]. The content of ginsenoside Rg1 in the stem and leaf of ginseng was 0.64% ± 0.004%, accounting for 27.15% of the total saponins in ginseng flowers [172]. Ginsenoside Rg1 is present in the stems, leaves, and flower buds of *Panax quinensis* and *Panax notoginseng* [180].

**FIGURE 4:**
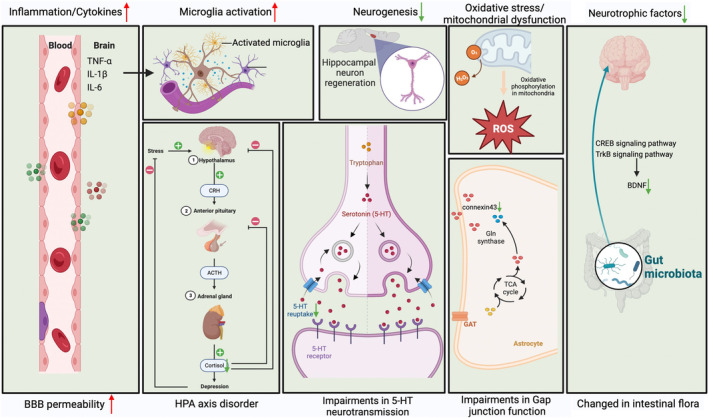
Mechanisms associated with depression pathogenesis. Elevated MAO‐A expression and decreased levels of 5‐HT and NE are considered major pathogenic factors for depression. After the body suffers from physiological or psychological stress, the HPA axis can be activated, leading to cascade effects. The hypothalamus secretes CRH, which stimulates the pituitary gland to release ACTH, subsequently triggering the adrenal cortex to release the final product COR. The decrease of BBB permeability may facilitate the entry of peripheral inflammatory factors into the CNS, leading to increased microglial activation. Depression is characterized by decreased neurogenesis in the hippocampus and altered synaptic remodeling across various brain regions, including the PFC. Empirically dependent synaptic remodeling and dendritic spine formation are affected by chronic stress and regulated via multiple signaling pathways, including BDNF. Mitochondrial dysfunction aggravates cell damage caused by oxidative stress. The imbalance of intestinal flora aggravates peripheral inflammation and may contribute to depression by affecting the neurotrophic factor pathway. Gap junction dysfunction in astrocytes, especially the decreased level of connexin43 protein, may be involved in depression pathogenesis and has the potential to become a new target for the treatment of depression. 5‐HT, serotonin; ACTH, adrenocorticotropic hormone; BBB, blood‐brain barrier; BDNF, brain‐derived neurotrophic factor; CNS, central nervous system; COR, cortisol; CRH, corticotropin‐releasing hormone; MAO‐A, type A monoamine oxidase; NE, norepinephrine; PFC, prefrontal cortex.

The synthesis of ginsenoside Rg1 has also been reported previously. Lu et al. found that a UDP‐glycosyltransferase from ginseng, UGTPg71A29, glycosylates the C20‐OH of Rh1 and transfers a portion of glucose to Rd. to produce ginsenosides Rg1 and Rb1 (Figure 4). The ectopic expression of UGTPg71A29 in *Saccharomyces cerevisiae* stably produced Rg1 and Rb1 in the presence of their corresponding substrates. UGTPg71A29 overexpression in transgenic ginseng cells significantly promoted the accumulation of Rg1, which was 3.2 times higher than that in controls [181]. UGTPg101, isolated from *Panax ginseng* , specifically glycosylates C6‐OH from protopanaxatriol to produce ginsenoside Rh1 and catalyzes ginsenoside F1 from protopanaxatriol. Ginsenoside Rg1 is subsequently generated from ginsenoside F1 [182]. Moreover, hesperidinase is prepared from *Penicillium* as raw material to selectively hydrolyze ginsenoside Re to produce ginsenoside Rg1 and L‐rhamnose [183].

### 3.3 Pharmacokinetics of Ginsenoside Rg1

Pharmacokinetic studies of Rg1 began in the 1980s. Recent advances in detection methods and in vivo and in vitro development technologies have led to new progress in understanding the pharmacokinetics of Rg1, primarily focusing on absorption mechanisms and metabolic transformations.

#### 3.3.1 Absorption

Odani, Tanizawa, Takino [184] used thin‐layer chromatography (TLC) to study the pharmacokinetics of Rg1 in rats. Rg1 content in the plasma was detected 15 min after oral administration of 100 mg/kg and reached a peak (0.9 μg/mL) at 30 min; however, it was not detected 6 h later. After oral administration for 15 min, 42.3% ± 1.6% and 35.6% ± 4.3% of Rg1 remained in the stomach and small intestine, respectively. After 30 min, most of the Rg1 advanced to the small intestine, at which point the large intestine contained 56.7% ± 5% of Rg1, and its oral bioavailability was only 1.9%. Following intravenous administration of 5 mg/kg in rats, the blood concentration was 8.9% ± 1.0 μg/mL at 2 min, which could not be detected after 60 min. Liang and Hua [185] found differences in the absorption of Rg1 across the entire digestive tract, with the highest absorption rate constant in the duodenum and the lowest in the stomach. In addition, the concentration of Rg1 affects its absorption, and the absorption rate constant at low and medium concentrations is greater than that at high concentrations. The absorption rate constant is not linear to the semi‐log concentration plot, suggesting that the absorption of Rg1 may involve other transport mechanisms beyond passive diffusion. Han and Fang [186] demonstrated significant gastric and intestinal first‐pass effects of Rg1 via in vivo drug administration in the digestive tract. With the maturation of cell models for studying drug absorption mechanisms, several researchers have used Caco‐2 cell models to investigate the uptake and transport of Rg1. The uptake of Rg1 exhibited time‐ and temperature‐dependent characteristics, demonstrating saturation rate processes with prolonged exposure. Experimental results on the concentration and pH dependence of Rg1 uptake vary across different laboratories, which is related to differences in cell culture conditions and dosages. Further confirmation is required to validate these findings [186, 187].

#### 3.3.2 Distribution

Odani, Tanizawa, Takino [184] found that in rats orally and intravenously administered Rg1, the liver and kidneys exhibited high drug concentrations, whereas no drug was detected in the brain. At 1.5 h after oral administration, peak drug concentrations were observed in all organs (except the brain). Intravenous administration resulted in a two‐phase elimination. Distribution characteristics of ginsenoside Rg1 in rats vary with different routes of administration. The blood concentration of ginsenoside Rg1 was measured 15 min after administration, and the mean maximum concentration (*C*_max_) was approximately 1 h. The Rg1 concentration in the liver was the highest 5 min after intravenous administration. Other tissues with high Rg1 concentrations included the kidneys, heart, lungs, spleen, and pancreas. However, after intravenous administration, almost no ginsenoside Rg1 was detected in the brain tissue. The area under the curve (AUC_0–*t*_) value of Rg1 in various tissues was in the order of kidney > liver > spleen > pancreas > heart > lung > brain, suggesting that ginsenoside Rg1 could not effectively cross the blood–brain barrier (BBB) during intravenous administration [188]. Other modes of administration may improve Rg1 distribution in the brain. Ginsenoside Rg1 is rapidly detected in the extracellular brain and cerebrospinal fluid of rats after subcutaneous administration, and the AUC in the medial PFC is significantly larger than that in the hippocampus [189]. Li et al. indicated that ginsenoside Rg1 crosses the BBB and is distributed in the cortex and hippocampus after administration to the abdominal cavity and inner ear [190, 191].

#### 3.3.3 Metabolism

Ginsenoside Rg1 metabolites have been detected in the stomach, large intestine, and cecum of rats. Thirty minutes after oral administration, three metabolites were detected in the stomach via TLC and ^13^C‐nuclear magnetic resonance identification. Two of these were identified as the 25‐position hydroxylated products of Rh1 and Rh1. The structure of the third metabolite was not determined because of instability. Additionally, Rh1 and F1 were found in the large intestine [192]. Wang et al. [193] confirmed that Rg1 is hydrolyzed to Rh1 and proginsenoside in the human intestine. Rh1 was obtained by removing the glucose moiety at C20 from Rg1, whereas F1 was obtained by removing the glucose moiety from the C6 side chain of Rg1. Similarly, the glucose at C6 and C20 of Rg1 was removed to obtain protopanaxatriol. Feng et al. [188] reported that Rg1 disappeared from the blood within 4 h after intravenous administration, with metabolites Rh1 and F1 detected in the blood 1 h post‐intravenous administration. Rh1 and F1 were completely absent from the blood after 24 h.

#### 3.3.4 Excretion

Feng et al. [188] demonstrated that Rg1 is primarily excreted in bile, and approximately 88.72% of Rg1 is excreted in its original form. Odani, Tanizawa, Takino [184] suggested that after the oral administration of 100 mg/kg Rg1, the cumulative excretion in urine and stool was 0.4% and 41.2% of the dose, respectively, and the cumulative excretion in bile was 1.1%. After the intravenous injection of 15 mg/kg Rg1, the cumulative bile excretion at 4 h was 57.2% of the dose, the cumulative urine excretion within 12 h was 23.5% of the dose, and approximately 80% of Rg1 was excreted via urine and bile. The oral recoveries of Rg1, Rh1, and protoginsenosides from bile, urine, and feces were > 70%. The recoveries of ginsenosides Rg1, Rh1, and protoginsenol in feces were 40.11%, 22.19%, and 22.88%, respectively, whereas the recoveries of Rg1 and Rh1 in bile were 6.88% and 0.09%, respectively. Only 0.04% of ginsenoside Rg1 was excreted in urine [194].

Therefore, ginsenoside Rg1 has low oral bioavailability. Rg1 cannot effectively reach the brain when administered intravenously but can reach the brain when administered subcutaneously, intraperitoneally, or intranasally. The main metabolites of ginsenoside Rg1 in the human intestine are ginsenoside Rh1 and protopanaxatriol, and the biliary pathway is primarily involved in their clearance.

## 4 Mechanisms of the Anti‐Depression Effect of Ginsenoside RG1

### 4.1 Reduction of Neuroinflammation

Rg1 exerts its antidepressant effects by inhibiting neuroinflammation. In a rat model of chronic restraint stress (CRS)‐induced depression, Rg1 restored standing and crossing counts in the open field test and reduced immobility time in the forced swimming test. Mechanistically, Rg1 inhibited the activation of microglia and significantly reduced the levels of pro‐inflammatory cytokines (TNF‐α, IL‐1β, and IL‐6) in the sera and hippocampus of CRS rats. Mitochondrial dysfunction has also been observed in depressed rats. In rats with CRS‐induced depression, ATP levels were significantly reduced and partially restored by Rg1 treatment. In addition, Rg1 attenuated mitochondrial damage and restored the mitochondrial aspect ratio. At the molecular level, Rg1 inhibits microglial activation and improves mitochondrial dysfunction by downregulating lncRNA GAS5, upregulating nuclear factor erythroid 2‐related factor 2 (NRF2), and suppressing cytokine signaling 3 [195].

Furthermore, Jiang et al. evaluated the antidepressant effect of Rg1 on chronic social defeat stress (CSDS)‐induced depressive behavior in mice. They discovered that long‐term administration of 20 or 40 mg/kg Rg1 significantly increased interaction time and sucrose intake and reduced immobility time in the sucrose preference test. To investigate the neuroimmune basis of the anti‐depressive‐like effects of Rg1, treatment with 20 or 40 mg/kg Rg1 reduced activated microglial morphology and lowered CSDS‐induced elevation of proinflammatory cytokines (TNF‐α, IL‐1β, and IL‐6) in CSDS mice. Additionally, treatment with 40 mg/kg Rg1 significantly reduced the expression of inducible nitric oxide synthase (iNOS) and cyclooxygenase‐2 (COX‐2). Additionally, Rg1 significantly blocked the increased expression of cleaved caspase‐3 and cleaved caspase‐9 in CSDS‐induced mice. The MAPK pathway in the hippocampus is also involved in the antidepressant effects of Rg1. Rg1 (20/40 mg/kg) treatment inhibited phosphorylated c‐Jun N‐terminal kinase (p‐JNK) and P38 expression, downregulated phosphorylated extracellular signal‐regulated kinase (p‐ERK)1/2/ERK1/2 expression, and decreased the ratio of p‐JNK/JNK in the MAPK signaling pathway in the hippocampi of mice subjected to CSDS. Moreover, Rg1 treatment reversed the reduction in hippocampal neurogenesis in adult CSDS mice, demonstrated by the restoration of doublecortin expression with 40 mg/kg Rg1 [196].

Similar conclusions have been drawn by Zhang et al. [197]. They found that treatment with 20 and 40 mg/kg Rg1 for 3 weeks improved depression‐related behaviors in CUMS rats, demonstrated by increased sucrose preference and reduced immobility activity and time in tail suspension and forced swimming experiments. The improvement effect of Rg1 on depressive symptoms in rats is related to CUMS‐induced neuroinflammation associated with the proinflammatory cytokine IL‐1β. Rg1 (40 mg/kg) also significantly inhibited the activation of the NF‐κB pathway and reduced the levels of nucleotide‐binding oligomerization domain‐like receptor family pyrin domain containing 3 (NLRP3) inflammasome, activating signal cointegrator‐1 and caspase‐1 in CUMS rats.

Rg1 also exerts antidepressant effects by regulating peripheral inflammatory responses. In the lipopolysaccharide (LPS)‐induced depression rat model, Rg1 effectively alleviated anorexia symptoms, partially reversed LPS‐induced weight loss, and increased sucrose preference and intake. Rg1 effectively eliminated the behavioral damage caused by neuroinflammation by blocking the LPS‐induced increase in plasma IL‐6 level, reversing the reduction of IL‐10 hippocampal transcription, and increasing the IL‐10 expression in the hippocampus. In addition, Rg1 inhibited the LPS‐associated elevation of iNOS expression and alleviated the microglial response, alongside behavioral improvement. Notably, these therapeutic effects do not depend on whether Rg1 directly penetrates the BBB (Table 2) [198].

**TABLE 2:** Mechanisms of antidepressant effect of ginsenoside Rg1.

Model	Administration and dosage	Efficacy	Mechanism	References
CRS‐induced male SD rats	20 mg/kg/day (*i.g*.), 28 days	Abrogate activation of microglial, decrease TNF‐α, IL‐1β, and IL‐6 levels, restore the expressions of SOCS3 and NRF2, recover the copy number of mtDNA, downregulate GAS5	Anti‐neuroinflammation, ameliorate mitochondrial dysfunction	Li et al. (2022a)
CSDS‐induced male C57BL/6J mice	20/40 mg/kg/day (*i.g*.), 28 days	Reduce the activated microglia morphology, decrease TNF‐α, IL‐1β, and IL‐6 levels, reduce iNOS and COX‐2 expression, block the increased cleaved caspase‐3 and caspase‐9 expression, inhibit p‐JNK and P38 expression, down‐regulate p‐ERK1/2/ERK1/2 expression, and decrease the ratio of p‐JNK/JNK	Anti‐neuroinflammation, promote neurogenesis	Jiang et al. (2020)
CUMS‐induced male SD rats	20/40 mg/kg/day (*i.p*.), 21 days	Decrease IL‐1β levels, inhibit the activation of the NF‐κB pathway, and reduce the levels of NLRP3 inflammasome, ASC‐1, and caspase‐1	Anti‐neuroinflammation	Zhang et al. (2019)
LPS‐induced male Wistar rats	10/30 mg/kg/day (*i.p*.), 3 days	Increase in plasma IL‐6 level, reverse the reduction of IL‐10 transcription in hippocampus, and increase the expression level of IL‐10 in hippocampus, inhibit iNOS expression and alleviate the microglial response	Anti‐neuroinflammation	Zheng et al. (2014)
CUMS‐induced Male Wistar rats	40 mg/kg/day (*i.p*.), 35 days	Increase the expression of miR‐134, enhance CREB protein expression and phosphorylation, and upregulate BDNF levels	Activate the CREB‐BDNF system	Yu et al. (2018)
CUMS‐induced Male Wistar rats	40 mg/kg/day (*i.p*.), 35 days	Increase CREB, BDNF, PSD‐95 expression, synaptophysin, inhibit the activation of microglia, reduce the levels of IL‐1β, IFN‐γ and TNF‐α, suppress the level of MDA, decrease caspase‐3 and caspase‐9 expression, increase expression of Bcl‐2	Protection of synaptic function, anti‐neuroinflammation, anti‐oxidative stress, anti‐apoptosis	Fan et al. (2018)
LPS‐induced male Wistar rats	40 mg/kg/day (*i.p*.), 14 days	Increase the number of synapses and vesicles, increase in dendritic spine density, increase in CREB phosphorylation and the expression of BDNF, inhibit glial activation and reduce the levels of IL‐1β, IL‐6, and IFN‐γ	Protection of synaptic function, anti‐neuroinflammation	Wang et al. (2023a)
CUMS‐induced Male Wistar rats	40 mg/kg/day (*i.p*.), 35 days	Increased ERK phosphorylation, attenuate the CUMS‐induced decrease in CREB phosphorylation, increase the protein levels of mature BDNF	Regulation of neurotrophic factors	Zhu et al. (2016)
CUMS‐induced Male Wistar rats	40 mg/kg/day (*i.p*.), 35 days	Upregulate BDNF expression, increase the phosphorylation of CREB, improve the decrease of PKA phosphorylation, prevent synaptic loss in the lateral amygdala	Regulation of neurotrophic factors	Liu et al. (2016)
CMS‐induced C57BL/6J mice	2.5, 5, 10, 20 mg/kg/day (*i.p*.), 14 days	Upregulate hippocampal BDNF signaling pathway, increase the expression of pERK1/2, enhanced the level of pCREB, decrease the level of serum corticosterone, reverse the decrease in dendritic spine density and hippocampal neurogenesis	Regulation of neurotrophic factors, protection of synaptic function	Jiang et al. (2012)
CUS‐induced male SD rats	20 mg/kg/day (*i.p*.), 46 days	Prevent the effect of CUS on gap junction communication function, increase the gap level between two adjacent astrocytes, and increase the expression of connexin43 protein	Regulation of gap junction function	Lou et al. (2020)
CUS‐induced male SD rats	5/10/20 mg/kg/day (*i.g*.), 28 days	Improve the functional activity of gap junctions, ameliorate ultrastructural alterations of astrocyte gap junctions, prevent the decrease of connexin43 protein and connexin43 mean fluorescence intensity	Regulation of gap junction function	Jin et al. (2017)
CORT‐treated primary astrocytes	0.1/1/10 μM, 1 h	Ameliorate the inhibition of astrocyte gap junction intercellular communication, increases connexin43 expression while decreasing connexin43 phosphorylation	Regulation of gap junction function	Xia et al. (2017)
CBX or Gap26‐induced male SD rats; CORT‐treated primary astrocytes	20 mg/kg/day (*i.g*.), 3 days; 0.1/1/10 μM, 1 h	Protect astrocyte against CORT‐induced cytotoxicity, improve the function of gap junctions, increase connexin43 levels	Protection of gap junction function	Xia et al. (2020)
CORT‐treated primary astrocytes	0.1/1/10 μM, 1 h	Alleviates the reduction of connexin43 protein, reverses CORT‐induced downregulation of connexin43 biosynthesis, decreases the degradation of connexin43 via ubiquitin‐proteasome degradation and autophagy‐lysosome degradation pathway	Regulate biosynthesis and degradation of connexin43	Wang et al. (2021a)
CUS‐induced male Wistar rats/LPS‐induced glial cells	40 mg/kg/day (*i.p*.), 31 days; 1 μM, 24 h	Reduce the increase of IL‐1β, IL‐2, IL‐6, IL‐18, TNF‐α and caspase‐1 levels, ameliorate inflammation‐induced glial gap junction dysfunction, reverse CUS induced upregulation of ubiquitination connexin43 levels	Anti‐neuroinflammation, regulate degradation of connexin43	Wang et al. (2021c)
CORT‐treated primary astrocytes	0.1/1/10 μM, 1 h	Increase phosphorylation of connexin43 at Tyr265 and Ser279 sites, upregulate total glutamine levels, glutamate content levels, and the ratio of glutamine to glutamate, decrease in glutamine synthetase activity, glutamate release and uptake	Regulation of glutamatergic system	Zhang et al. (2022)
CUMS‐induced male SD rats	20/40 mg/kg/day (*i.g*.), 21 days	Reduce glutamate and aspartate levels, increase the concentration of taurine and gamma amino butyric acid	Regulation of glutamatergic system	Wu et al. (2012)
CUMS‐induced male rats	40 mg/kg/day (*i.p*.), 28 days	Increase SOD and GSH‐pX, reduce MDA and NO, inhibit 8‐oHdG expression, decrease the levels of NOX1 and NOX4, reduce IL‐1β, IFN‐γ, and TNF‐α levels	Inhibition of oxidative stress	Li et al. (2020)
CUMS‐induced male SD rats	5/10/20 mg/kg/day (*i.g*.), 3 days	Reduce the serum corticosterone levels, increase the serum testosterone level, increase the protein level of glucocorticoid receptor, and the number of androgen receptor‐positive cells	Regulation of HPA and HPG axis	Mou et al. (2017)

Abbreviations: ASC‐1, activating signal cointegrator‐1; BDNF, brain‐derived neurotrophic factor; CBX, carbenoxolone; CMS, chronic mild stress; CORT, glucocorticoid corticosterone; COX2, cycloase‐2; CREB, CAMP‐response element binding protein; CRS, chronic restraint stress; CSDS, chronic social defeat stress; CUMS, chronic unpredictable mild stress; CUS, chronic unpredictable stress; ERK, extracellular signal‐regulated kinase; GAS5, growth arresting‐specific 5; GSH‐pX, glutathione peroxidase; HPA, hypothalamic–pituitary–adrenal; HPG, hypothalamic–pituitary–gonadal; i.g., intragastric; i.p., intraperitoneal; iNOS, inducible nitric oxide synthase; LPS, lipopolysaccharide; MDA, malondialdehyde; NLRP3, nucleotide‐binding oligomerization domain‐like receptor family pyrin domain containing 3; NO, nitric oxide; NOX1, NADPH oxidase 1; NRF2, nuclear factor erythroid 2‐related factor 2; p‐ERK, phosphorylated extracellular signal‐regulated kinase; p‐JNK, phosphorylated c‐Jun N‐terminal kinase; SOCS3, suppressor of cytokine signaling 3; SOD, superoxide dismutase.

### 4.2 Regulation of Synaptic Function

Ginsenoside Rg1 alleviates depression by regulating synaptic function. In a rat model of CUMS‐induced depression, chronic administration of ginsenoside 40 mg/kg/d Rg1 significantly increased sucrose consumption. In open‐field experiments, pretreatment with Rg1 significantly increased the number of crossings and immobility time. Transmission electron microscopy revealed that chronic pretreatment with Rg1 improved synaptic reduction in the basolateral amygdala (BLA) induced by CUMS in rats. Ginsenoside Rg1 pretreatment also ameliorated mitochondrial degradation and cytoplasmic vacuolation in CUMS‐induced rats, and the reduction in ultrastructural abnormalities induced by ginsenoside Rg1 indicated its potential neuroprotective effects. Considering the potential of miR‐134 to regulate synaptic structural plasticity, Yu et al. [199] confirmed the effect of Rg1 pretreatment on miR‐134 levels and found that Rg1 chronic pretreatment significantly increased the miR‐134 expression in the BLA, indicating that the antidepressant effect of Rg1 in depressed rats may be related to the miR‐134 signaling pathway in the BLA. Additionally, ginsenoside Rg1 enhanced CREB protein expression and phosphorylation and upregulated BDNF levels in the BLA of CUMS‐induced rats. This finding suggested that ginsenoside Rg1 may also exert antidepressant effects by activating the CREB‐BDNF system.

In the same depression rat model, Fan et al. similarly concluded that ginsenoside Rg1 exhibited a protective effect against CUMS‐induced depressive‐like behavior. In the sucrose preference test, pretreatment with 40 mg/kg Rg1 reversed reduced sucrose consumption in CUMS‐exposed rats, reduced immobility, and increased swimming in the forced swim test. Long‐term exposure to CUMS significantly reduced the density of dendritic spines in the ventral medial prefrontal cortex (vmPFC) region of rats, and ginsenoside Rg1 pretreatment significantly ameliorated this CUMS‐induced change in dendritic spine density. Transmission electron microscopy revealed that chronic preconditioning with ginsenoside Rg1 significantly ameliorated synaptic loss in vmPFC neurons. These morphological results suggest that the ability of ginsenoside Rg1 to protect the vmPFC neuronal structures may be an important mechanism underlying its antidepressive effects. Ginsenoside Rg1 significantly upregulated the CUMS‐induced downregulation of synapse‐related proteins (CREB, BDNF, PSD‐95, and synaptophysin), further verifying the protective effect of Rg1 on synapses. Ginsenoside Rg1, when chronically pretreated in CUMS rats, protected synapses, inhibited excessive microglial activation, reduced pro‐inflammatory cytokine levels (IL‐1β, IFN‐γ and TNF‐α), suppressed malondialdehyde (MDA) levels induced by CUMS, and prevented alterations in caspase‐3, caspase‐9, and Bcl‐2 expression in the vmPFC induced by CUMS exposure [200].

Thus, combining ginsenoside Rg1 with voluntary running may synergistically alleviate depressive symptoms. Wang et al. indicated that synergistic treatment prevented the reduction in sucrose consumption in the sucrose preference test, reversed the increase in immobility in the forced swimming test, and increased the central area residence time and total open‐field distance in LPS rats. Treatment with ginsenoside Rg1, coupled with voluntary wheel running, significantly increased synapse and vesicle numbers in the hippocampus, accompanied by an increase in dendritic spine density. In addition, ginsenoside Rg1 treatment alone prevented the LPS‐induced decrease in CREB phosphorylation in the hippocampus and vmPFC and increased BDNF expression in the hippocampus. Ginsenoside Rg1 alone or combined treatment inhibited glial activation and reduced pro‐inflammatory cytokine levels (IL‐1β, IL‐6, and IFN‐γ) in the vmPFC [201].

### 4.3 Regulation of Neurotrophic Factors

One possible mechanism by which ginsenoside Rg1 improves depressive symptoms is the activation of the BDNF pathway. Zhu et al. demonstrated that long‐term administration of ginsenoside 40 mg/kg Rg1 significantly improved depressive‐like behavior in rats, as assessed by sucrose preference and forced swimming tests. Chronic preconditioning with ginsenoside Rg1 significantly increased ERK phosphorylation in the PFC, and pretreatment with ginsenoside Rg1 significantly attenuated the CUMS‐induced decrease in CREB phosphorylation in the PFC. Furthermore, researchers examined mature BDNF in the PFC to assess its potential involvement in the antidepressant effects of ginsenoside Rg1. Long‐term pre‐administration of ginsenoside Rg1 significantly increased mature BDNF protein levels in the PFC [202].

To further determine the possible involvement of neurotrophic factors in the antidepressant effects of ginsenoside Rg1, Liu et al. examined BDNF expression in the lateral amygdala (LA) region. Immunofluorescence assays showed that BDNF expression in the LA region decreased after 5 weeks of CUMS exposure, whereas pretreatment with 40 mg/kg/day ginsenoside Rg1 significantly improved the downregulation of BDNF expression induced by CUMS. Additionally, ginsenoside Rg1 increased CREB phosphorylation in the LA region. Moreover, the decrease in PKA phosphorylation induced by CUMS was significantly ameliorated after ginsenoside Rg1 treatment. Transmission electron microscopy revealed that ginsenoside Rg1 pretreatment significantly prevented CUMS‐induced synaptic loss in the LA of CUMS exposure [203].

Similarly, in a chronic mild stress (CMS) depression model, Rg1 upregulated the hippocampal BDNF signaling pathway. It also increased pERK1/2 expression, enhanced the level of pCREB in the hippocampal tissue, and decreased the level of serum corticosterone. Additionally, Rg1 reversed the decrease in dendritic spine density and hippocampal neurogenesis caused by CMS [204].

### 4.4 Regulation of Gap Junction Function

Recent studies have confirmed that the ginsenoside Rg1 exerts antidepressant effects by regulating gap junction function and connexin43 expression. Rg1 prevents the effect of chronic unpredictable stress (CUS) on gap junction communication function by widening the gap between two adjacent astrocytes in the hippocampal CA1 region induced by CUS and upregulating connexin43 protein expression in rats [205]. Jin et al. [206] also demonstrated that long‐term Gg1 treatment of CUS‐exposed rats can significantly improve the ultrastructure of astrocyte gap junctions and prevent a decrease in dye diffusion in the PFC, suggesting beneficial effects on the functional activity of brain gap junction channels. Xia et al. [207] discovered Rg1 preconditioning significantly improved gap‐junction intercellular communication in the PFC and hippocampal corticosteroid‐treated astrocytes, which was associated with the upregulation of connexin43 expression and downregulation of connexin43 phosphorylation. These findings further confirm the role of Rg1 in increasing astrocyte gap junction intercellular communication, which offers a potential therapeutic avenue for depression.

Previous studies have shown that Rg1 preconditioning can significantly improve connexin43‐gap connectivity in astrocytes in both in vivo and in vitro models of depression, thus providing a new way to ameliorate depression [208]. Wang et al. [209] confirmed that Rg1 counteracts the inhibitory effect of CORT on connexin43 biosynthesis by upregulating connexin43 mRNA expression. Additionally, Rg1 can reverse the upregulation of connexin43 ubiquitin‐proteasome degradation in CORT‐induced astrocytes, reduce LC3‐II/LC3‐I values in the PFC and hippocampal astrocytes, and improve connexin43 reduction level, preventing CORT‐induced accelerated degradation of astrocyte connexin43. Wang et al. [210] also demonstrated that Rg1 normalized the decrease in the fluorescence diffusion area induced by inflammatory cytokines, suggesting that Rg1 can ameliorate inflammation‐induced glial gap junction dysfunction. Rg1 treatment also reverses CUS‐induced upregulation of ubiquitination connexin43 levels, indicating that Rg1 may reduce depression by inhibiting connexin43 ubiquitination, thereby improving neuroinflammation.

### 4.5 Regulation of the Glutamatergic System

Disorders of the glutamate system may be a downstream mechanism of depression induced by gap junction channel dysfunction. In a CORT‐induced glutamate system dysfunction depression model, Rg1 improved the glutaminergic system dysfunction of astrocytes and reversed the CORT‐induced downregulation of total glutamine, glutamate, and glutamine‐to‐glutamate ratios. In addition, Rg1 ameliorated the CORT‐induced increase in glutamine synthetase activity. Furthermore, Rg1 treatment reversed CORT‐induced increases in glutamate release and uptake, suggesting that Rg1 can improve CORT‐induced glutaminergic system dysfunction [211].

Ginsenoside Rg1 also regulates glutamate systems in depression animal models. Rg1 intervention significantly increased sucrose consumption and the sucrose preference rate of CUMS rats and the horizontal and vertical motor scores of depressed rats. Mechanistic studies have confirmed that Rg1 intervention significantly reduces glutamate (Glu) and aspartate (Asp) levels and increases the concentration of taurine (Tau) and gamma amino butyric acid (GABA) in the hippocampus of stressed rats. Glu is an important excitatory substance in the HPA axis. The HPA axis is activated during stress, causing accumulation of Glu and resulting in hippocampal toxicity [212]. CRH is sensitive to Asp concentration, and GABA and Tau can inhibit hyperactivity of the HPA axis [213]. Ginsenoside Rg1 effectively ameliorates depressive symptoms by mitigating the toxic effects of Glu and Asp accumulation in the hippocampus after chronic stress in rats, increasing inhibitory amino acids, such as GABA and Tau levels, and decreasing the ratio of excitatory/inhibitory amino acids [214].

### 4.6 Additional Mechanisms Underlying the Anti‐Depression Effect of Ginsenoside Rg1

Inhibition of oxidative stress may contribute to the antidepressant effects of ginsenoside Rg1. Li et al. found that CUMS exposure induced a decrease in oxidase activity in the CA1 region of the hippocampus while increasing the level of oxidative stress. Mitochondrial oxidative stress is enhanced, such as increased MitoSOX levels. Moreover, these effects were accompanied by oxidative DNA damage as indicated by the DNA base damage marker 8‐oHdG. However, chronic preconditioning with ginsenoside Rg1 inhibited CUMS‐induced oxidative stress and DNA damage. In addition, ginsenoside Rg1 downregulated the CUMS‐induced upregulation of nicotinamide adenine dinucleotide phosphate (NADPH) oxidase, (NOX) 1, and NOX4. These findings suggest that the antidepressant effects of ginsenoside Rg1 may be due, in part, to a reduction in oxidative stress and may be mediated via the NOX1/NOX4 pathway [215].

Ginsenoside Rg1 exerts antidepressant activity by regulating the HPA and hypothalamic–pituitary–gonadal (HPG) axes. Mou et al. [55] confirmed that Rg1 has significant antidepressant activity in acute and chronic stress models. Experiments showed that Rg1 significantly reduced the resting time of mice in the forced swimming and tail suspension tests. Rg1 improved depression symptoms, such as helplessness, anhedonia, and sleep disruption, in the CUMS model by adjusting the HPA and HPG axes and improved behavioral abnormalities in the gonadectomized (GDX) treatment model. Rg1 decreases serum corticosterone levels, increases serum testosterone levels, elevates PFC and hippocampal GR protein levels, downregulates serum corticosterone levels, and increases androgen receptor protein levels in the PFC.

**FIGURE 5:**
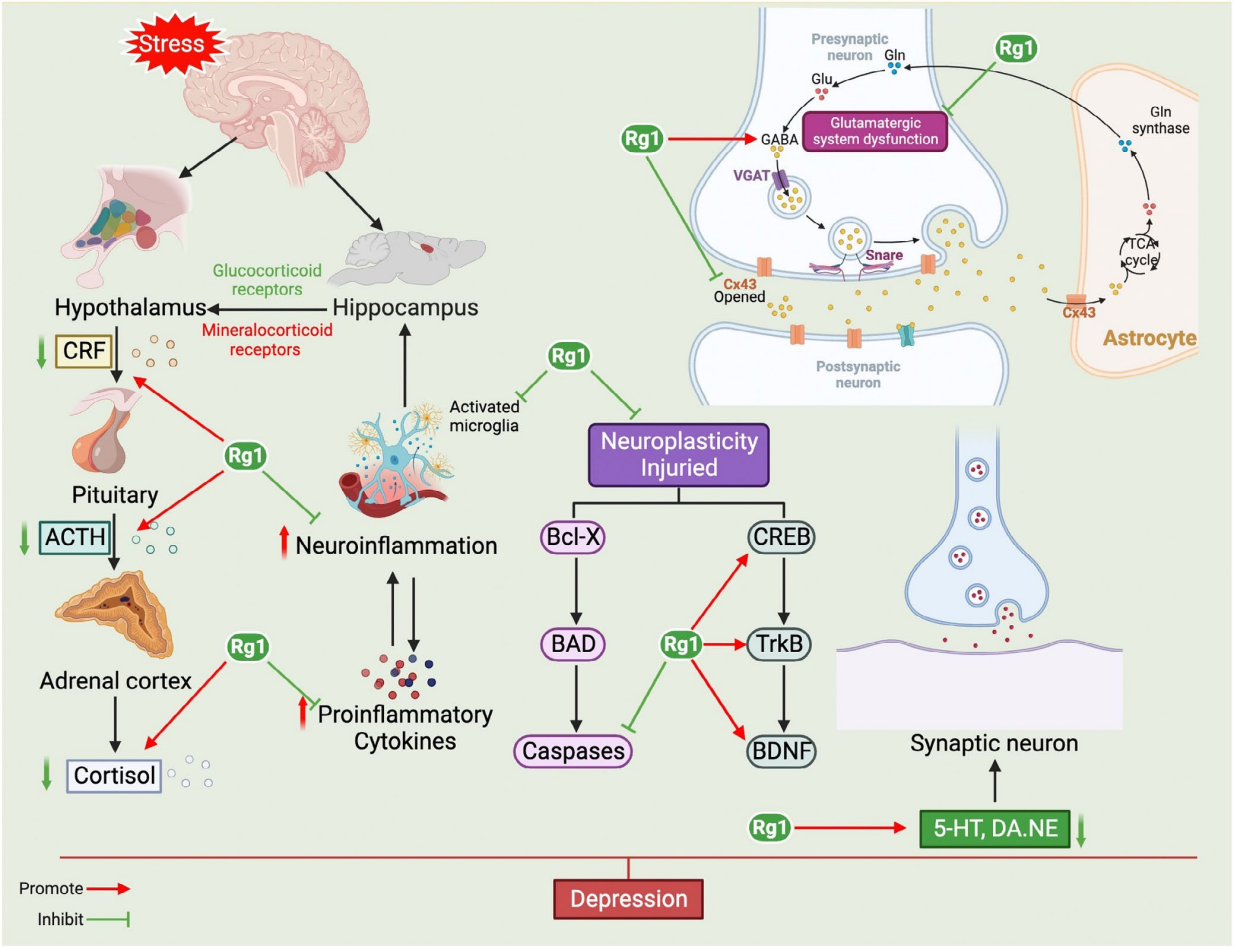
Mechanism of the antidepressant effect of ginsenoside Rg1. Ginsenoside Rg1 inhibits the hyperfunction of the hypothalamic–pituitary–adrenal (HPA) axis, increases glucocorticoid receptor expression, and decreases the level of corticosterone. Rg1 protects the gap junction function of astrocytes and inhibits inflammation by increasing the level of connexin 43. Rg1 increases synaptic‐related protein expression in the hippocampus, cortex, and amygdala and regulates synaptic plasticity. Rg1 can also destroy the inflammatory loop, prevent microglial over‐activation, and reduce the level of pro‐inflammatory factors. Meanwhile, Rg1 can regulate the level of oxidative stress and inhibit neural apoptosis.

Ginsenoside Rg1 selectively promotes the growth of beneficial bacteria, such as *Bifidobacterium*, *Lactobacillus*, and *Akkermansia*, in the gut. It also inhibits the overgrowth of harmful bacteria, such as *Clostridium* and *Enterococcus* [216]. Ginsenoside Rg1‐induced changes in gut microbiota composition are associated with therapeutic effects. In addition, ginsenoside Rg1 enhances intestinal barrier function and alleviates metabolic endotoxemia by modulating the gut microbiota in diet‐induced obese mice [217]. Moreover, the prebiotic‐like effects of ginsenoside Rg1 on gut microbiota contribute to its protective role against metabolic disorders. Rg1 also inhibited the metabolism of intestinal microbial‐derived tryptophan and decreased the levels of 5‐HT, 5‐HTR1B, and 5‐HTR2A. Although Rg1 modulates tryptophan metabolism and the serotonergic system by regulating the gut flora, research in depression models remains scarce (Figure 5).

## 5 Potential Targets Involved in the Protection of RG1 Against Depression

In addition to the pharmacological mechanisms of Rg1 in depression treatment described above, we screened its targets using network pharmacology. We initiated network pharmacology analysis to identify the Rg1 “drug‐disease” target in depression. Using SwissTargetPrediction (http://www.swisstargetprediction.ch/), we obtained all possible Rg1 targets, followed by screening depression‐associated genes (score > 1.0) from the GeneCards database (https://www.genecards.org/). Gene Ontology and Kyoto Encyclopedia of Genes and Genomes (KEGG) analyses were performed (https://david.ncifcrf.gov/), highlighting pathways, such as protein kinase B (Akt), phosphatidylinositol 3‐kinase (PI3K), and PI3K‐Akt signaling pathways.

**FIGURE 6:**
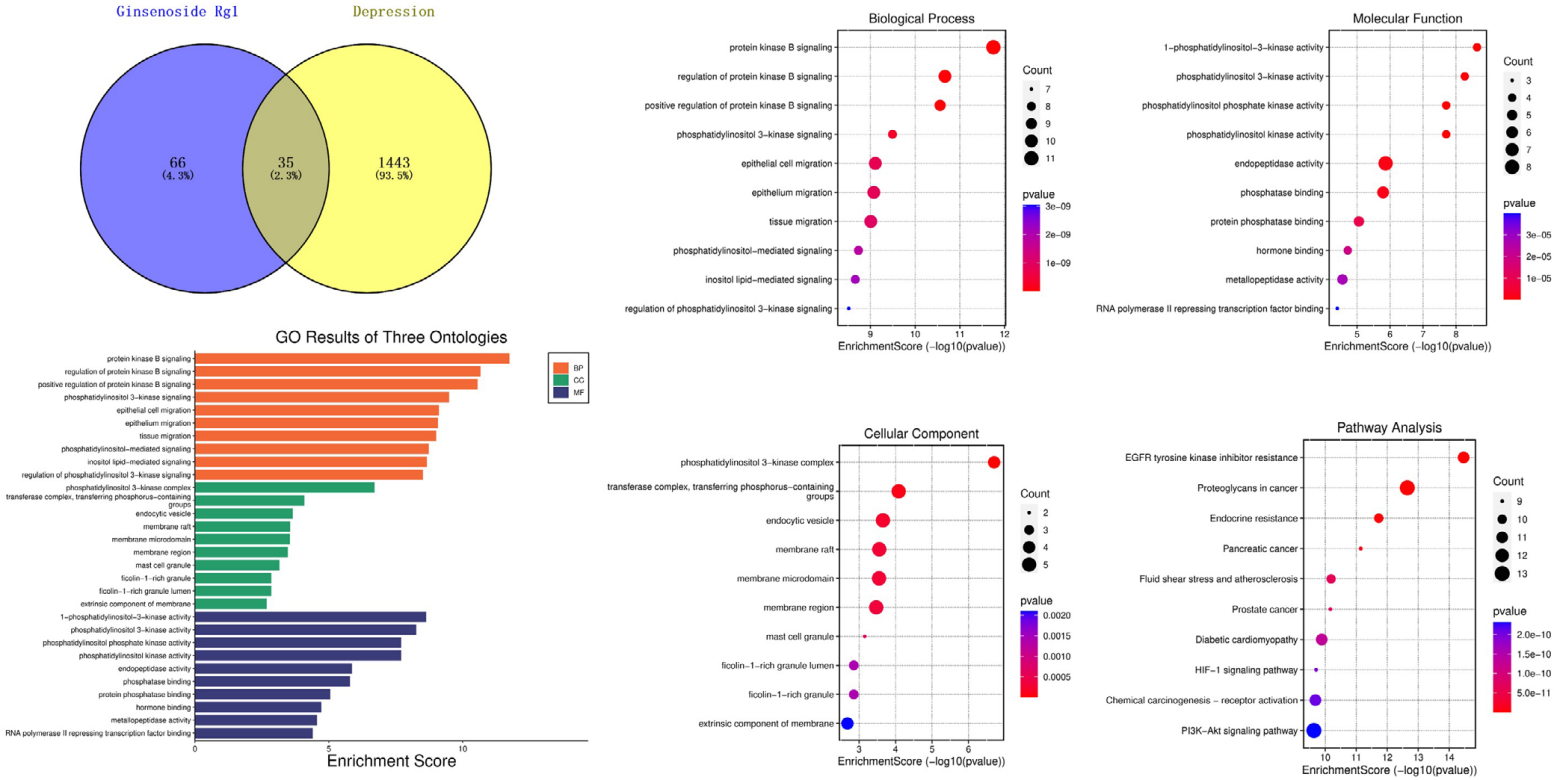
“Drug‐disease” targets of ginsenoside Rg1 and depression; gene ontology (GO; Top 10) and Kyoto Encyclopedia of Genes and Genomes (KEGG; Top 10) enrichment analysis of common targets.

**FIGURE 7:**
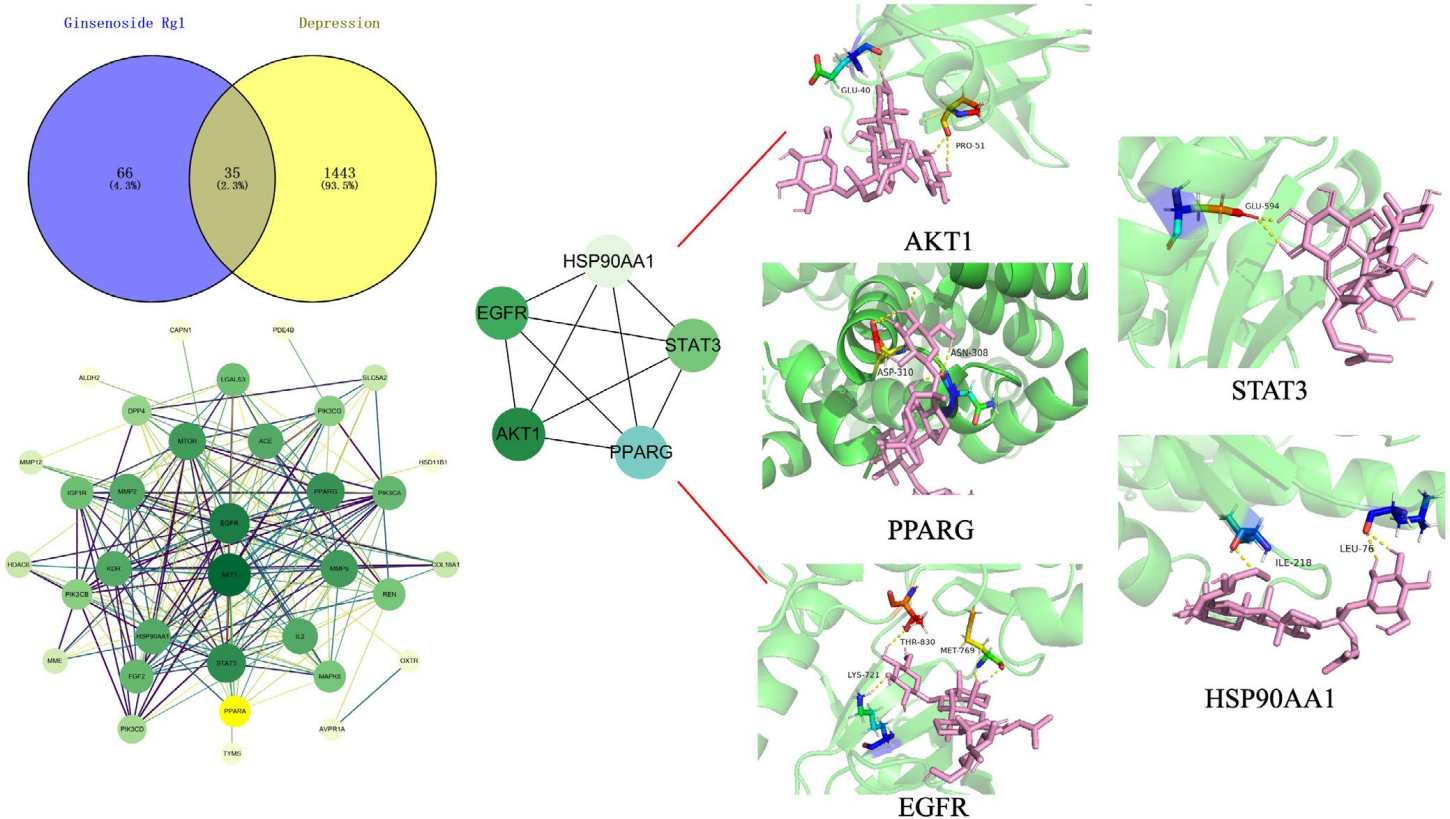
Potential targets of Rg1 and molecular docking of Rg1 against depression. Venn diagram and drug‐targets‐disease network of Rg1 action targets and depression‐related genes. There are 101 Rg1 action targets and 1478 depression‐related genes, 35 of which are common targets. Five hub genes (*AKT1, STAT3, EGFR, PPARG*, and *HSP90AA1*) were extracted from the PPI network and subjected to molecular docking analysis. Their respective binding energies were −1.43, −0.21, −1.85, −1.82, and −2.19 kcal/mol.

Moreover, we imported the obtained targets into Cytoscape 3.9 software and combined them with the STRING database (https://cn.string‐db.org/) to complete a protein–protein interaction (PPI) network. Finally, hub genes in the PPI network were selected based on the above evidence, and molecular docking was performed using AutoDockTools 1.5.6 and PyMOL software combined with the PDB database (https://www.rcsb.org/) to identify the reliability of the targets obtained via network pharmacology. A total of 101 potential targets of Rg1 and 1478 depressive‐related targets were identified, with 35 overlapping targets. Molecular docking was conducted with selected intersecting targets, including AKT1, STAT3, EGFR, PPARG, and HSP90AA1, all demonstrating high docking efficacy. This highlights the potential biological role of Rg1 in depression treatment, indicating that Rg1 is a promising therapeutic agent (Figures 6 and 7).

## 6 Perspective and Conclusions

Emerging from experimental research and pharmacological network analysis, ginsenoside Rg1 has been identified as a promising candidate for the therapeutic management of depression. Its potential therapeutic efficacy is underpinned by an accumulating body of preclinical evidence, which delineates its capacity to modulate a spectrum of molecular pathways central to the pathophysiology of depressive disorders. Notably, ginsenoside Rg1 exhibits several mechanisms of action, including: (i) Neurotransmitter modulation: Rg1 has been shown to augment the levels of neurotransmitters such as serotonin and dopamine, which are frequently depleted in patients with depression. (ii) Neuroinflammation regulation: Chronic neuroinflammation is a pivotal factor in the etiology of depression. Rg1 is capable of mitigating neuroinflammation by modulating the expression of inflammatory cytokines and suppressing the activation of microglial cells. (iii) Neuroprotection: Rg1 demonstrates neuroprotective effects by preventing apoptosis and fostering neurogenesis, both of which are essential for the preservation of brain health and the recovery from depressive states. (iv) Receptor pathway interaction: Rg1 interacts with various receptor pathways, including those associated with the Akt, PI3K complex, and PI3K‐Akt signaling, which may underpin its antidepressant effects. Pharmacological network analysis indicates that ginsenoside Rg1 may exhibit synergistic effects when combined with other bioactive compounds, thereby potentially enhancing its therapeutic efficacy. Furthermore, the natural origin of ginsenoside Rg1, coupled with its relatively favorable safety profile, positions it as an appealing candidate for the development of innovative antidepressant therapeutic agents.

However, further research is required in the following areas. Firstly, the antidepressant mechanism of Rg1 requires further clarification. Research on the anti‐inflammation, neurogenesis, and gap junction function of astrocytes is insufficient. Further investigation is required to reveal the exact neuroprotective mechanisms of Rg1. Secondly, there is insufficient research on structural modifications of Rg1. As a plant‐derived active compound, Rg1 possesses several inherent defects, including low bioavailability and insufficient blood concentrations. In addition, investigating the delivery modes of Rg1 is a crucial future research direction. Yu et al. [218] constructed a novel brain‐targeted drug delivery system based on borneol‐modified polyethylene glycol graphene oxide, which can assist Rg1 in the effective use of brain‐targeted drug delivery and provide a new strategy for the treatment of depression. Thirdly, the limitations of ginsenoside Rg1 in its antidepressant activity and the evaluation models employed for its efficacy are extensive, including the construction of depression models, the administration of drug dosages, and the timing of treatment. These aspects necessitate further standardized design in subsequent animal studies to expedite the application of ginsenoside Rg1 in clinical trials. Finally, the lack of controlled clinical trials remains a major barrier to the application of Rg1 in clinical practice. In the future, we anticipate conducting controlled clinical trials of Rg1 based on sufficient in vivo studies.

In conclusion, Rg1 may play an antidepressant role via various mechanisms, including anti‐inflammation, synaptic function protection, promotion of neurogenesis and BNDF release, improvement of astrocytes gap junction function, and HPA axis and antioxidative stress regulation. A substantial body of experimental research validates the effectiveness of Rg1 in treating depression, with molecular docking analysis supporting its promising application prospects in the treatment of depression. Despite challenges in pharmacokinetic studies and animal experiments, current research still has limitations. Owing to the developmental potential and research challenges of Rg1, we anticipate its swift adoption as a widely used antidepressant in clinical practice.

## Conflicts of Interest

The authors declare no conflicts of interest.

## Data Availability

The authors have nothing to report.
